# Biofilm in Genital Ecosystem: A Potential Risk Factor for *Chlamydia trachomatis* Infection

**DOI:** 10.1155/2019/1672109

**Published:** 2019-01-22

**Authors:** Simone Filardo, Marisa Di Pietro, Giulia Tranquilli, Rosa Sessa

**Affiliations:** Department of Public Health and Infectious Diseases, ‘Sapienza' University, Rome 00185, Italy

## Abstract

In healthy women, the cervicovaginal microbiota is mostly populated by *Lactobacillus* spp., the main host defense factor of the female genital tract. In addition to Lactobacilli, other microorganisms populate the cervicovaginal microbiota, like *Candida* spp. and *Gardnerella vaginalis*. The overgrowth of *Candida* spp. or *G. vaginalis*, known as biofilm-producing microorganisms in the genital ecosystem, may lead to microbial dysbiosis that increases the risk of acquiring sexually transmitted infections, like *Chlamydia trachomatis*. *C. trachomatis*, the leading cause of bacterial sexually transmitted diseases, is still considered an important public health problem worldwide because of the impact of asymptomatic infections on long-term reproductive sequelae, including pelvic inflammatory disease and infertility. The aim of our study was to investigate the interaction between *C. trachomatis* and the biofilm produced by *Candida albicans* or *Gardnerella vaginalis*, evaluating whether the biofilm can harbor *C. trachomatis* and influence its survival as well as its infectious properties. In order to do so, we developed an *in vitro* coculture transwell-based biofilm model. Our findings proved, for the first time, that *C. trachomatis*, an intracellular obligate pathogen, survived, for up to 72 hours after exposure, inside the biofilm produced by *C. albicans* or *G. vaginalis*, retaining its infectious properties, as evidenced by the typical chlamydial inclusions observed in the cell monolayer (chlamydial inclusion-forming units at 72 h: 9255 ± 1139 and 9873 ± 1015, respectively). In conclusion, our results suggest that the biofilm related to *Candida* or *Gardnerella* genital infections may act as a reservoir of *C. trachomatis* and, thus, contribute to the transmission of the infection in the population as well as to its dissemination into the upper genital tract, increasing the risk of developing severe reproductive sequelae.

## 1. Introduction

In healthy women, the cervicovaginal microbiota is known to play a fundamental role in the defense of the female genital tract against potential infectious threats. A microbiota dominated by *Lactobacillus* spp. is classically associated with a healthy genital ecosystem [1, 2], since it has been shown to inhibit the growth of potential pathogens by competing for nutrients, releasing antimicrobial compounds, activating immune system pathways, and maintaining a low vaginal pH through the production of lactic acid [3–5].

In addition to Lactobacilli, other microorganisms populate the cervicovaginal microbiota, including *Candida* spp. and *Gardnerella vaginalis.* The overgrowth of *Candida* spp. or *G. vaginalis* and the depletion of *Lactobacillus* spp. may lead to a microbial imbalance that can precipitate in a genital infection [2].

Amongst genital infections, vulvovaginal candidiasis and bacterial vaginosis are the most frequent disorders in reproductive women, contributing to 90% of all cases of vaginitis [6]. *Candida albicans* is known as the most common cause of vulvovaginal candidiasis; in fact, almost 75% of the female population experience an episode at least once in their lifetime, 50% of whom experience at least a second episode, and 5–10% of all women experience recurrent vulvovaginal candidiasis [7]. Also, bacterial vaginosis, responsible for more than 60% of all cases of vaginitis in women of childbearing age, where *Gardnerella vaginalis* is the predominant bacterial species [8], has a 60% recurrence rate in the 12 months after metronidazole treatment [9].

The main virulence trait associated with recurrent *C. albicans* or *G. vaginalis* genital infections is the formation of a biofilm [8, 10], characterized by complex microbial communities attached to a substrate and surrounded by an extracellular matrix [11].

Importantly, biofilm-related infections have a unique clinical significance due to the tendency of embedded pathogens to harbor resistance against host defense factors and antimicrobial agents [12]. Indeed, it is well known that the biofilm may be responsible for treatment failure as well as recurrence of genital infections [13, 14]. In this regard, the observation of the presence of *Candida* on intrauterine devices (IUDs) removed from patients with genital infections is particularly interesting, suggesting that biofilm formation on IUDs might be an important risk factor for recurrent candidiasis [15]. Furthermore, the use of IUDs seems to also increase the risk for bacterial vaginosis [16].

In recent years, evidence that the *C. albicans* biofilm is capable of retaining herpes simplex virus type-1 without altering its infectivity led to the compelling hypothesis that the biofilm could potentially be a reservoir of sexually transmitted pathogens [17]. Consequently, it is tempting to speculate that the biofilm produced by *C. albicans* or *G. vaginalis* may also increase the risk of acquiring *Chlamydia trachomatis*, known as the leading cause of bacterial sexually transmitted diseases.

*C. trachomatis* is an obligate intracellular pathogen with a distinctive developmental cycle characterized by the extracellular infectious elementary body (EB), that invades the host cell, and the intracellular replicative reticulate body (RB), responsible for the multiplication within the host [18].

*C. trachomatis* is still considered an important public health problem worldwide because of the impact of asymptomatic infections (90%) on long-term reproductive sequelae, including pelvic inflammatory disease (PID) [19]. Particularly important, an increased risk of PID has also been observed in IUD users affected by *C. trachomatis* infection [20, 21].

Therefore, the aim of our study was to investigate the interaction between *C. trachomatis* and the biofilm produced by *C. albicans* or *G. vaginalis*. Specifically, we analyzed whether the biofilm can harbor *C. trachomatis* and influence its survival as well as its infectious properties.

## 2. Materials and Methods

### 2.1. Microbial Strains

The strains of *Candida albicans* (ATCC 10231), *Gardnerella vaginalis* (ATCC 4944), and *Chlamydia trachomatis* serovar D/UW-3Cx (ATCC VR-885), used in this study, were obtained from the American Type Culture Collection (ATCC), USA.

### 2.2. Cell Culture

The human epithelial HeLa-229 cell line (ATCC CCL-2.1) from the cervix adenocarcinoma was cultured at 37°C in Dulbecco's modified Eagle's medium (DMEM, EuroClone), supplemented with 10% (v/v) heat-inactivated fetal calf serum (FCS), in a humidified atmosphere with 5% CO_2_.

### 2.3. Propagation and Titration of *Chlamydia trachomatis*

*C. trachomatis* was propagated in HeLa-229 cells and grown in DMEM supplemented with 10% FCS, as previously described [22]. The infectious titer (inclusion-forming units per mL (IFUs/mL)) was assessed by the immunofluorescence assay. In brief, subconfluent HeLa-229 cell monolayers grown on glass coverslips in 24-well plates were infected with 10-fold serial dilutions of bacterial stock, incubated for 48 hours at 37°C, fixed with methanol, and stained with fluorescein isothiocyanate-conjugated monoclonal antibody anti-*C. trachomatis* LPS (IMAGEN Chlamydia kit, Oxoid). The total number of *C. trachomatis* IFUs was enumerated by counting all microscope fields using a fluorescence microscope (400x magnification).

### 2.4. Biofilm Formation

**Figure 1:**
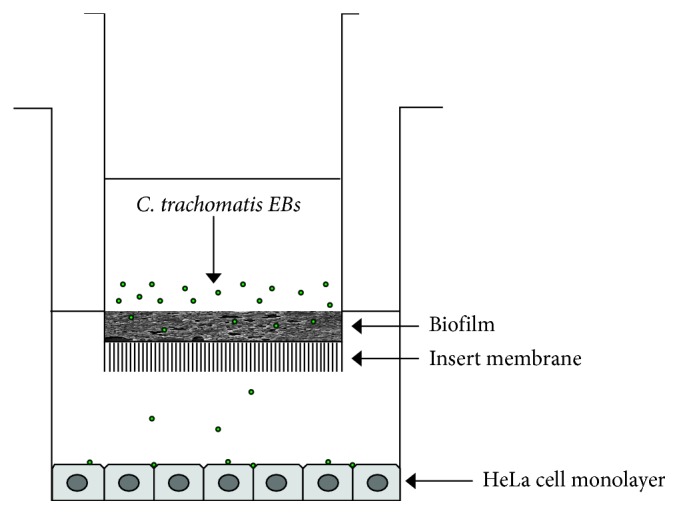
Experimental setup. *C. albicans* or *G. vaginalis* biofilms were produced in the upper chamber of transwell coculture systems. After 24 hours, *C. trachomatis* (as indicated by the arrow) was inoculated onto the biofilm, and epithelial cell monolayers, grown on glass coverslips, were seeded in the lower chamber.

Transwell coculture systems (0.4 *µ*m pore size, polyester membrane, Corning) were used for *C. albicans* or *G. vaginalis* biofilm formation (Figure 1). In brief, *C. albicans* was grown overnight at 37°C in yeast peptone dextrose, harvested, washed twice with phosphate-buffered saline (PBS), and then resuspended at a concentration of 1 × 10^6^ cells/mL in RPMI supplemented with 10% FCS. Next, 500 *µ*L of *C. albicans* cell suspension was seeded in triplicate on the insert membranes placed in a 24-well tissue plate and incubated at 37°C to allow the biofilm production.

*G. vaginalis* was grown in brain heart infusion supplemented with 2% (w/w) gelatin, 0.5% (w/w) yeast extract, and 0.1% (w/w) starch for 24 hours at 37°C with 10% CO_2_ and, then, diluted to a final concentration of approximately 10^7^ colony-forming units (CFUs)/mL. Next, 500 *µ*L of *G. vaginalis* suspension was seeded in triplicate on the insert membranes and incubated as described above to allow the biofilm production.

The next day, the culture medium was removed, and the inserts were washed once with PBS to eliminate nonadherent microbial cells. Biofilm formation was assessed by the crystal violet assay.

### 2.5. Exposure of Biofilms to *C. trachomatis*

After 24 hours, 100 *µ*L of *C. trachomatis* (2.5 × 10^6^ EBs) in DMEM with 10% FCS was added onto *Candida* or *Gardnerella* biofilms, and at the same time, subconfluent HeLa-229 cell monolayers, grown on glass coverslips, were seeded into the lower chamber (Figure 1). The transwell systems were, then, incubated at 37°C and 5% CO_2_. Twelve, 24, 48, and 72 hours later, the cell monolayers were removed from the lower chamber, and the presence of *C. trachomatis* was determined by the immunofluorescence assay, as previously described [22].

In other experiments, the inoculum of *C. trachomatis* and the cell suspensions of *C. albicans* or *G. vaginalis* were simultaneously added on the insert membranes, and after 48 hours, the amount of biofilm was quantified by the crystal violet assay.

### 2.6. Crystal Violet Assay

Inserts containing *Candida* or *Gardnerella* biofilms or controls (medium only) were washed 3 times with PBS and then air-dried for 5 min. Following fixation by 96% methanol for 20 min, the samples were stained with 1% crystal violet (CV) solution for 5 min. After three washes with distilled water, 1 mL of 33% acetic acid was added to each well. The optical densities (ODs) were measured at 594 nm 10 min later by using a microplate reader (Biotek).

### 2.7. Statistical Analysis

All values are expressed as mean ± standard deviation (SD) of three replicates from three independent experiments. Comparison of means was performed by using a two-tailed Student's *t*-test for independent samples. A value of *P* ≤ 0.05 was considered statistically significant.

## 3. Results

As expected, *C. albicans* and *G. vaginalis* produced biofilms on the transwell membranes 24 hours after the inoculation, as evidenced by the crystal violet assay (OD_595 nm_ values: 1.1 ± 0.3 and 2.2 ± 0.9, respectively).

**Figure 2:**
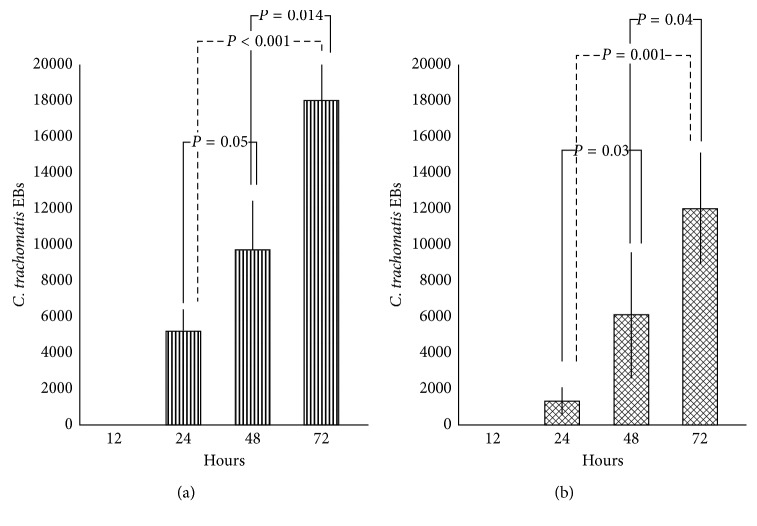
Release of *C. trachomatis* EBs from biofilms. Number of *C. trachomatis* EBs released from *C. albicans* (a) or *G. vaginalis* (b) biofilms detected at 24-hour intervals up to 72 hours from initial exposure.

**Figure 3:**
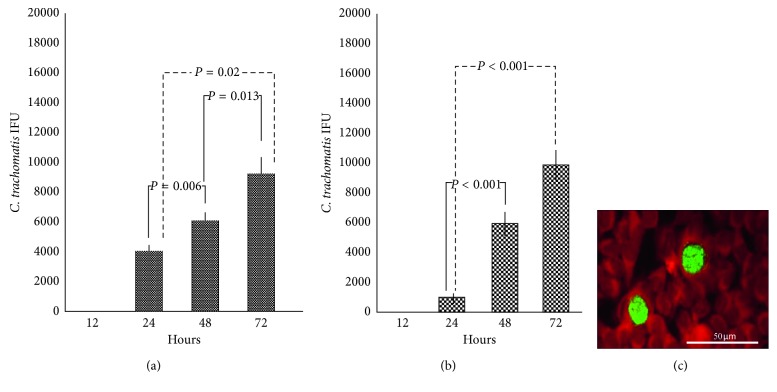
Infection of HeLa cell monolayers following *C. trachomatis* release from biofilms. Infectivity of *C. trachomatis* EBs released from *C. albicans* (a) or *G. vaginalis* (b) biofilms detected at 24-hour intervals up to 72 hours from initial exposure. (c) Immunohistological staining of *C. trachomatis* inclusions visualized in HeLa cell monolayers by fluorescence microscopy (400x magnification).

Then, we evidenced that the biofilm produced by *C. albicans* or *G. vaginalis* was able to retain *C. trachomatis* for up to 12 hours, as evidenced by the absence of EBs in cell monolayers removed from the lower chamber of the transwell system. Interestingly, the number of chlamydial EBs released from the *Candida* or *Gardnerella* biofilm increased significantly in a time-dependent manner (Table 1; Figures 2(a) and 2(b)), and more importantly, *C. trachomatis* was still able to infect and replicate within host cells (Table 2; Figures 3(a) and 3(b)). In fact, typical inclusions were found in cell monolayers removed from the lower chamber of transwell systems, confirming the ability of *C. trachomatis* to complete its developmental cycle (Figure 3(c)).

**Figure 4:**
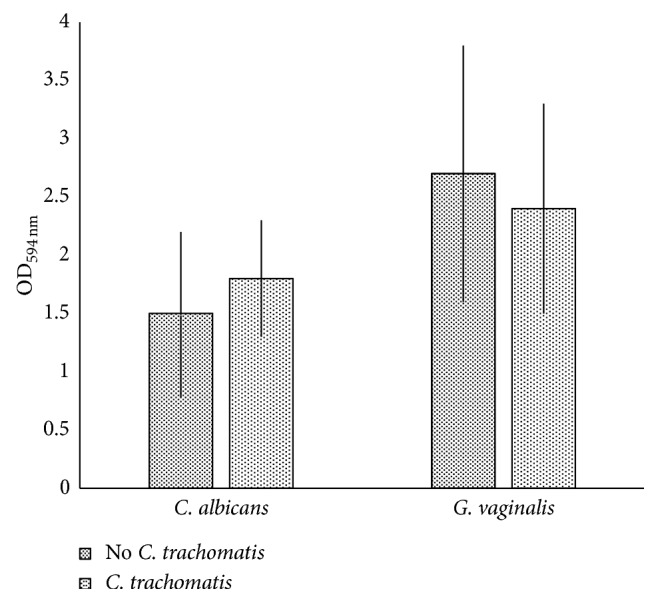
*Candida* and *Gardnerella* biofilm formation in the presence or absence of *C. trachomatis*. OD_594 nm_ values were measured 48 hours after incubation of *C. albicans* and *G. vaginalis* in the presence or absence of *C. trachomatis*.

Lastly, we observed that the biofilm produced by *C. albicans* or *G. vaginalis* was unaffected by the presence of *C. trachomatis*. Indeed, no significant differences in the biofilm OD_595 nm_ values were observed between the combinations of *C. trachomatis* with *C. albicans* (1.8 ± 0.5) or *G. vaginalis* (2.4 ± 0.9) and the microbial species alone (controls) (*C. albicans*: 1.5 ± 0.7, *P* = 0.58; *G. vaginalis*: 2.7 ± 1.1, *P* = 0.73) (Figure 4).

## 4. Discussion

This is the first study investigating the interaction between the biofilm produced by *C. albicans* or *G. vaginalis* and *C. trachomatis*, since previously published reports on biofilms were exclusively focused on pathogens involved in infections of medical devices, such as catheters, prostheses, and heart valves [23, 24].

For reaching our goal, we developed an *in vitro* transwell-based model which allowed us to monitor the effects of biofilm exposure to *C. trachomatis* over time. The biofilm was produced in the upper chamber, while the epithelial cell monolayer was placed in the lower one; such a system closely resembles a more physiological microenvironment and allowed us to investigate *C. trachomatis* interaction with *Candida* or *Gardnerella* biofilm. Our *in vitro* model also mimics the biofilm growing on artificial surfaces, including IUDs, known risk factors for recurrent genital infections [15, 16]. More importantly, our *in vitro* model was essential to directly observe the ability of the biofilm to enclose *C. trachomatis* EBs while gradually releasing them, producing clear images of chlamydial inclusions visualized by fluorescence microscopy. By contrast, chlamydial inclusions were not easily visualized when the biofilm was produced on cell monolayers grown on traditional cell-culture microplates.

The main result of our study is the ability of *C. trachomatis* to survive inside the biofilm produced by *C. albicans* or *G. vaginalis.* Specifically, we demonstrated that *C. trachomatis* survived within the biofilm, retaining its infectious properties, for up to 72 hours after exposure, as evidenced by the numerous typical inclusions observed in the cell monolayers removed from the transwell system. As a result, both *Candida* and *Gardnerella* biofilms may provide a protective niche for the survival of *C. trachomatis*, reducing its antibiotic susceptibility as well as favoring the evasion of the host immune system. In fact, it is well known that microorganisms encased in biofilms are generally well protected against environmental stresses, antibiotics, and disinfectants, as well as the host immune system, and thus, they are extremely difficult to eradicate [25].

Several clinical treatment failures have been reported concerning *C. trachomatis* genital infections [4, 26, 27], and a possible explanation may lie in the presence of a potential biofilm protecting *C. trachomatis*. Such hypothesis may also be suggested by the evidence that microbial dysbiosis in a cervicovaginal ecosystem, usually characterized by the overgrowth of biofilm-producing microorganisms, may contribute to the acquisition of *C. trachomatis* infection [1, 3].

In our study, the observation that *C. trachomatis*, released from the biofilm, was able to infect and replicate within epithelial cells is extremely intriguing. In the literature, in fact, there is a plethora of studies demonstrating that biofilm dispersion/detachment is most likely to play a significant role in a long-term colonization, dissemination, and transmission of pathogens [28, 29].

Therefore, the continuous release of *C. trachomatis* infectious EBs following the biofilm dispersion/detachment is of particular pathological importance as this phenomenon may contribute to the dissemination of this pathogen to the upper genital tract, leading to severe reproductive sequelae; notably, up to 26% of women with *C. trachomatis* infection develop PID, nearly 10% of women with PID have ectopic pregnancy, and up to 38% of women with recurrent episodes of PID may become infertile [19, 30].

## 5. Conclusions

In conclusion, our findings could have important clinical implications since the biofilm associated with *Candida* or *Gardnerella* genital infections may act as a chlamydial reservoir contributing to the transmission of *C. trachomatis* in the population, alongside its dissemination in the female upper genital tract. The survival of *C. trachomatis* within the biofilm may also help to explain the increased risk of PID in *C. trachomatis*-infected women who also use IUDs, where the biofilm formation is more likely to happen.

In the future, it will be interesting to translate our results in clinical studies to confirm that the biofilm produced by microorganisms colonizing the genital ecosystem may be considered as a risk factor for *C. trachomatis* infection.

## Figures and Tables

**Table 1:** *C. trachomatis* EBs released from *C. albicans* or *G. vaginalis* biofilms 24, 48, and 72 hours after exposure.

Biofilm	Number of *C. trachomatis* EBs		
	24 h	48 h	72 h
*C. albicans*	5200 ± 1210	9700 ± 2750	18000 ± 2234
*G. vaginalis*	1300 ± 797	6100 ± 3513	12000 ± 3137

EB: elementary body.

**Table 2:** Number of *C. trachomatis* IFUs observed on HeLa cell monolayers 24, 48, and 72 hours after exposure.

Biofilm	Number of *C. trachomatis* IFUs		
	24 h	48 h	72 h
*C. albicans*	4100 ± 375	6122 ± 557	9255 ± 1139
*G. vaginalis*	982 ± 298	5925 ± 807	9873 ± 1015

IFU: inclusion-forming unit.

## Data Availability

The data used to support the findings of this study are included within the article.
